# Thermodynamic Equilibrium Study of Ash Transformation during Entrained Flow Conversion of Agricultural Biomass Focusing on the Potential Extraction of Valuable Si and K–P Compounds via Condensation from the Gas Phase

**DOI:** 10.1021/acsomega.5c01125

**Published:** 2025-04-25

**Authors:** Samarthkumar Pachchigar, Thomas Karl Hannl, Marcus Öhman

**Affiliations:** † Energy Engineering, Department of Engineering Sciences and Mathematics, Luleå University of Technology, SE-97187 Luleå, Sweden; ‡ BEST—Bioenergy and Sustainable Technologies, GmbH, Inffeldgasse 21b, AT-8010 Graz, Austria; § Institute of Chemical and Energy Engineering, BOKU University, Muthgasse 107/I, 1190 Vienna, Austria

## Abstract

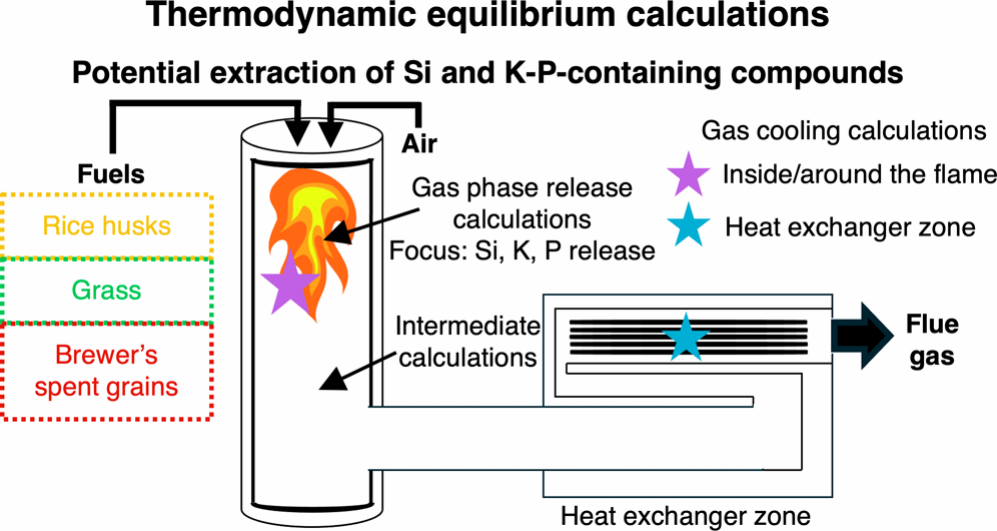

Agricultural biomass is today largely underutilized in combustion and gasification processes because of the abundant supply of other easier-to-process biomass fuels. These biomass types generally have a moderate to high ash content comprising valuable elements, such as Si, K, and P, which can lead to ash-related operational problems. The high share of Si, K, and P in agricultural biomass assortments also has a significant economic value. These elements are usually retained in the coarse or fly ash fractions. Extracting valuable Si- and K–P-containing compounds with high purity from these ash fractions often requires further postprocessing steps, which increases operational costs. Therefore, a potential novel design concept could be to control the combustion/gasification processes so that Si, K, and P can be extracted by condensation from the flue/hot gases at a quality that implies added value instead of extra costs. This work aims to identify the possibilities of extracting valuable Si and K–P compounds from the hot gases generated during entrained flow conditions via stepwise controlled condensation in the close-flame regions or heat exchanger zone. Thermodynamic equilibrium calculations were performed by employing the databases (GTOX and SGPS) in FactSage 8.0 software. The calculations were performed under varying conditions ,i.e., temperatures, atmospheres, and fuel compositions. The selected fuels were rice husks (Si-rich), brewer’s spent grains (P–Si-rich with moderate to minor amounts of Ca, Mg, and K), and grass (K–Si-rich with moderate amounts of Ca, Mg, and P). The results indicate that the high-temperature formation of the valuable Si compounds, such as SiC (s) and Si_2_N_2_O (s), would require an inert atmosphere during both the release and cooling stages. Moreover, a high Si/P molar ratio is needed to form valuable Si-containing compounds. The predicted K-bearing phosphates during the gas cooling near the burner zone were formed in the same temperature range as Ca-, Mg-, and Si-containing compounds with all of the fuels. The results obtained by this study can guide experimental research on the practical extraction of Si and K–P compounds from different types of agricultural biomass during thermochemical conversion in entrained flow conditions.

## 1 Introduction

Biomass fuels comprise a wide range of primarily solid fuels with large variations in their inherent heating value and ash composition.^1^ Common solid biomass assortments used for thermochemical conversion are woody and agricultural biomass. The viability of using these different biomass types in thermal conversion processes depends largely on their ash composition.^2,3^ The impact of the ash composition may range from beneficial aspects like catalytic gas reforming in gasification processes^4^ to detrimental aspects such as slagging, fine particle emission, and agglomeration.^5^ Especially for agricultural biomass, these detrimental aspects may induce severe operational problems due to their ash composition. The primary reasons for increased ash-related operational problems with agricultural biomass are the formation of melt and emission of corrosive compounds caused by higher shares of K, Si, S, Cl, and P in the fuel compared to woody biomass.^6−8^

Although the elevated share of these ash-forming elements in most agricultural biomasses may pose a risk for the operation of the thermochemical conversion process, it also enables the implementation of resource recovery strategies. The high Si, K, and P content in these biomass assortments is of potentially significant economic value, which is essentially unexploited as ash is regarded as a waste that entails handling and disposal costs. Si-containing components are raw materials for advanced materials applications in, e.g., metal, battery, and electronics industries and for filters, ceramic insulation, high-strength concrete, and solar cells.^9^ The valuable Si-containing compounds for advanced materials applications are SiC (s), Si_3_N_4_ (s), Si_2_N_2_O (s), and SiO_2_ (s).^9,10^ On the other hand, K and P are essential macronutrients required in large quantities for plant growth and are commonly used in fertilizer production.^11^ Several K- and P-containing compounds, such as KH_2_PO_4_,^12^ CaKPO_4_, KMgPO_4_, (K_2_, Ca, Mg)_2_P_2_O_7_, and (K_2_, Ca, Mg)(PO_3_)_2_, have been reported to be found in produced ash fractions during combustion of agricultural biomasses.^13^ These compounds are potentially valued for their solubility and nutrient availability, making them interesting for use in agricultural fertilizers. The strategies to efficiently recover these potentially valuable elements depend on the actual fuel ash composition and the possibility of selectively extracting these elements in the form of valuable compounds. Currently, the research on extracting such elements in the form of valuable compounds from the combustion/gasification of agricultural biomass is mostly focused on the post-treatment of collected ash,^14−16^ which can be expensive. One possible alternative to this approach could be the direct extraction of Si and K–P compounds with high purity via subsequent condensation from the gases formed along with energy conversion, i.e., by sequentially separating the valuable pure compounds via removing various ash fractions at different temperatures. This approach relies on a process that operates at temperatures high enough to volatilize the targeted compounds and selective condensation of these compounds.

Entrained flow conversion operates at higher temperatures than other technologies (e.g., fixed bed and fluidized bed conversion), which offers a possibility for a high gas-phase release (i.e., volatilization) of certain elements in the flame,^17^ which can later be condensed on cooled surfaces inside or around the flame in the burner zone and/or in the heat exchanger zone. According to previous studies,^18,19^ the fuel particle can experience comparatively higher temperatures inside the flame than the furnace temperature during the entrained flow operation. Moreover, the fuel particles are exposed to reducing conditions inside the flame. Both of these aspects could enhance the formation of volatile ash compounds during the initial fuel conversion stage. The subsequent condensation of volatilized ash compounds is influenced by the gas atmosphere in the burner and heat exchanger zones, the interaction of gaseous elements, and the temperature regime.^6,20^ Investigating all these relevant variables in practice requires a complex experimental and analytical setup to determine all the sensitive parameters. For instance, previous studies conducted on lab-scale entrained flow combustion of rice husks, grass, and brewer’s spent grains (BSG) at 1200 and 1450 °C reported a limited gas-phase release of Si (<1%), while minor to moderate gas-phase release of K and P for all the fuels.^21,22^ These results highlight the need to evaluate the conditions for a higher gas-phase release of fuel-inherent Si, K, and P. A capable alternative for this determination is thermodynamic equilibrium calculations (TECs), which can assist in determining the sensitivity to measure variation in such processes. These model calculations can be conducted for various fuels and process parameters and facilitate the design of future experiments and practical setups.

The aim of this work is to employ thermodynamic equilibrium calculations for assessing the potential of extracting valuable Si- and K–P-containing compounds by investigating conditions that enhance the gas-phase release of ash-forming elements and subsequent condensation under entrained flow conversion conditions. For this purpose, three different fuels were analyzed by implementing the parameters defining the fuel conversion and condensation stages in the model. The fuels were chosen to represent agricultural biomasses with different ash compositions, i.e., rice husks (i.e., Si-rich), brewer’s spent grains (i.e., BSG, P–Si-rich with moderate to minor amounts of Ca, Mg, and K), which is a residue from a beer-brewing industry, and grass (i.e., K–Si-rich with moderate amounts of Ca, Mg, and P). The results obtained in this work could contribute to the design of entrained flow processes targeting the recovery of different ash-forming elements in the form of valuable compounds.

## 2 Methodology

### 2.1 Fuel

The thermodynamic calculations were done based on the fuel characterization of three different agricultural biomass, namely, rice husks, brewer’s spent grains (i.e., BSG), and grass. Rice husks containing a high share of Si were obtained from Humlegårdens Ekolager AB, located near Stockholm, Sweden. Rice husks were previously dedusted and steam-sterilized at 130 °C by the manufacturer. BSG was a residue from beer production and had a high share of P and Si, with moderate to minor amounts of Ca, Mg, and K in the ash. BSG was obtained from beer-brewing company Spendrups Bryggeri AB, Grängesberg, Sweden. Grass containing a high share of K and Si with a moderate amount of Ca, Mg, and P was delivered by a German energy supplier, Brennpunkt Energie GmbH, located in Ruderatshofen, Germany. The characteristics of used fuels are presented in Table 1.

**Table 1:** Fuel Characteristics of the Selected Fuels^d^

	rice husks	BSG	grass
Proximate Analysis			
moisture content (wt % w.b.)^a^	5.5	4.5	4.9
ash content (wt % d.b.)^b^	12.3 ± 0.1	3.9 ± 0.1	8.4 ± 0.1
Ultimate Analysis (wt % d.b.)^b^			
C	41.7 ± 0.2	50.1 ± 0.3	43.2 ± 0.3
H	5.4 ± 0.2	6.6 ± 0.4	6.0 ± 0.5
N	0.6 ± 0	4.5 ± 0	2.6 ± 0
O^c^	40	34.9	39.8
Elemental Analysis (mmol/kg d.b.)^b^			
Na	1.3 ± 0.0	4.6 ± 0.1	17.3 ± 0.2
K	59.0 ± 0.3	26.7 ± 0.3	536.2 ± 3.9
Ca	23.7 ± 0.4	84.9 ± 0.1	184.8 ± 0.9
Mg	17.1 ± 0.1	84.9 ± 0.6	128.1 ± 0.6
Fe	1.1 ± 0.1	6.1 ± 0.1	11.1 ± 0.3
Zn	-	2.0 ± 0.0	-
Al	10.5 ± 0.3	1.7 ± 0.0	32.9 ± 0.6
Si	1841.4 ± 23.2	169.0 ± 10.2	317.3 ± 5.1
P	24.3 ± 0.3	216.0 ± 0.9	121.5 ± 0.8
S	13.0 ± 0.2	98.2 ± 1.8	63.9 ± 1.3
Cl	6.0 ± 0.1	11.8 ± 0.2	110.0 ± 4.7
total (mmol/kg_d.b.)	1997.4 ± 25.0	705.9 ± 14.3	1523.1 ± 18.3

a w.b.: wet basis, d.b.

b Dry basis.

c By difference, - below detection limit.

d The fuel characteristics of rice husks were adapted with permission from Pachchigar et al.,^21^*Energy Fuels*. **2024,***38,* 14, 13278–13294. Copyright 2024 American Chemical Society. Available under CC BY 4.0 license. The fuel characteristics of BSG and grass were adapted with permission from Pachchigar et al.,^22^*Energy Fuels*. **2025,***39,* 2, 1384–1400. Copyright 2025 American Chemical Society. Available under CC BY 4.0 license.

The ash-forming elements were analyzed by inductively coupled plasma atomic emission spectroscopy (ICP-AES) according to ISO 16967 (i.e., for Na, K, Ca, Mg, Fe, Al, Si, and P) and ion chromatography (IC) according to ISO 16994 (i.e., for S and Cl). The CHN content of the fuels was analyzed by an EURO EA 3000 elemental analyzer according to ISO 16948. The ash content analysis was performed according to ISO 18122.

### 2.2 Thermodynamic Equilibrium Calculations

The thermodynamic equilibrium calculations (TECs) were performed in the software FactSage 8.0, based on the minimization of the total Gibbs free energy of the system with a global equilibrium approach, i.e., uniform chemical potential and pressure throughout the system. The databases used during the calculations were GTOX (stoichiometric compounds, molten and solid solutions, and gas species) as the primary database and SGPS (stoichiometric compounds and gas species) as the secondary database. This combination of databases was used to provide more data for gas-phase compounds present in SGPS and the high compatibility between GTOX and SGPS.^23^ For duplicate data sources of compounds, the GTOX compound was prioritized. The selected databases and solution models for the TECs can be seen in Table 2.

**Table 2:** Selected Elements, Databases, and Solution Models Used for TECs in FactSage 8.0

elements	
H, C, N, O, Na, K, Ca, Mg, Fe, Zn, Al, Si, P, S, Cl	
Database—GTOX (Solution Models)	
SLAG: slag phase containing oxides, metals, sulfides, and fluorides	FSPA: feldspar (Al, Fe)(K, Na)Si_3_O_8_
ALFA: (K, Na)_2_SO_4_, (Ca, Mg)SO_4_	HEXA: (K, Na)_2_SO_4_, (Ca, Mg)O
CAS: (Ca, Mg, Mn)S with solubility for FeS	OLIV: olivine (Mg, Fe)_2_SiO_4_
C2SA: (Ca, Mg)_2_(Si, P)O_4_	SIOM: SiO_2_ with solubility for AlPO_4_
CLIN: CLINO_PYROXENE (Ca, Mg)_2_Si_2_O_6_	WOLL: wollastonite (Ca, Mg)SiO_3_
CMP: CaP_2_MgO_7_	stoichiometric solid compounds
gas compounds	
Database—SGPS (Stoichiometric Compounds)	
gas compounds	stoichiometric solid compounds

**Figure 1:**
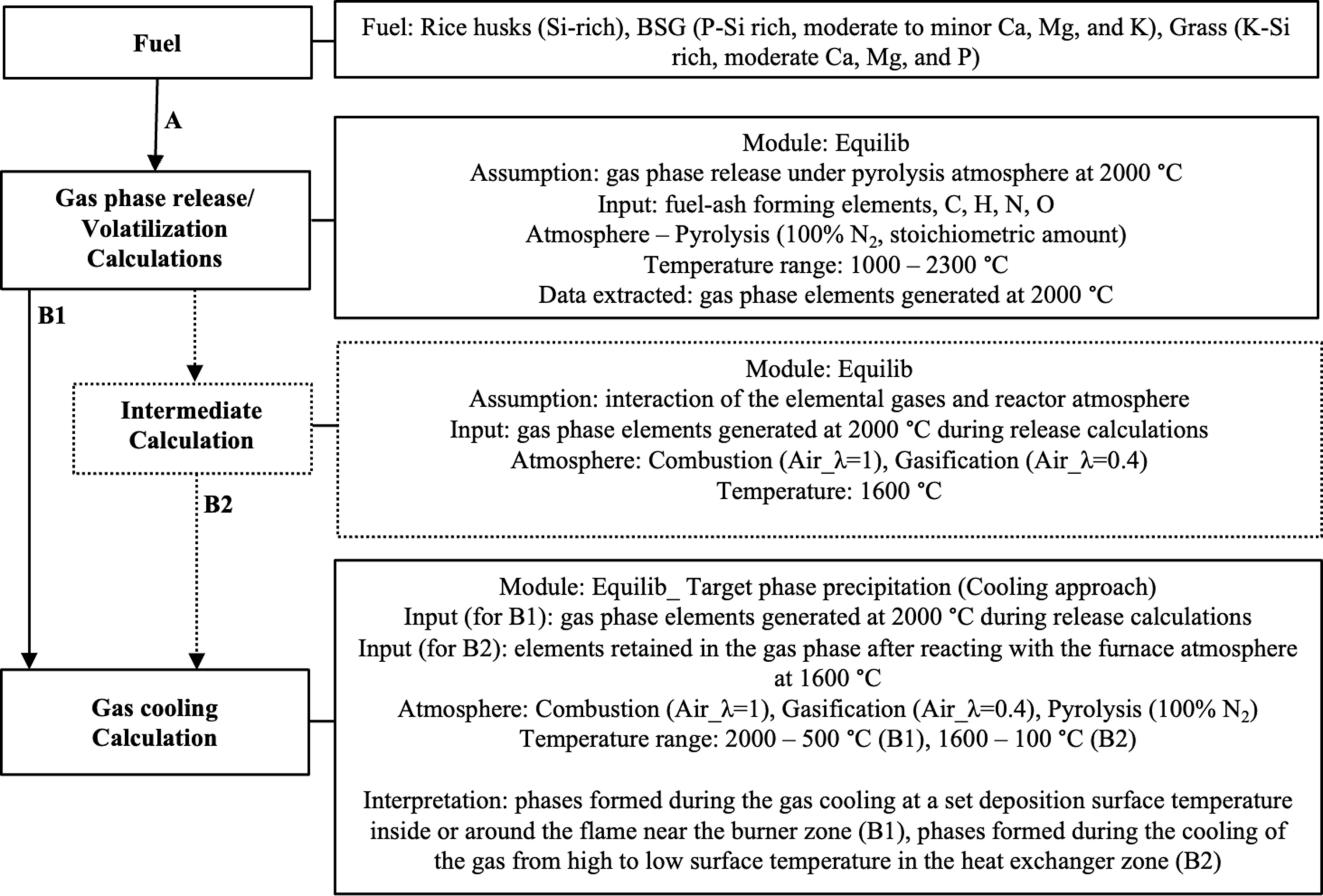
Schematic representation of the calculation approach in FactSage 8.0.

The modeling of entrained flow conditions in FactSage was carried out based on two consecutive events occurring throughout the process, namely, the volatilization of gaseous species and subsequent condensation of compounds. Primarily, the fuel particles are exposed to the flame and experience a pyrolysis atmosphere. This conversion step leads to the volatilization of ash-forming elements depending on the temperature inside the flame. Figure 1 describes the calculation approaches applied to identify the gas-phase release and potential quality of the condensed compounds under equilibrium conditions. The gas-phase release calculations (A, Figure 1) were performed in the pyrolysis atmosphere (i.e., N_2_) for each fuel between 1000 and 2300 °C. This temperature range of 1000 to 2300 °C was selected to determine the influence of flame temperature on the volatilization of ash-forming elements. The lower limit (i.e., 1000 °C) represents the point at which the volatilization of target elements like K and P either initiated or reached moderate levels, according to the calculations. In contrast, 2300 °C allows for the study of extreme high-temperature effects, including volatilization of Si, which predominantly occurs at elevated temperatures. The input data for the release part of the calculation refers to the ash-forming elements (i.e., Na, K, Ca, Mg, Fe, Zn, Al, Si, P, S, and Cl), C, H, N, and O content within each fuel as well as N_2_ addition with a molar gas-to-ash ratio around 100 for rice husks and grass and around 340 for BSG. This molar ratio of gas to fuel ash refers to the amount calculated for the theoretical amount of air required for complete combustion (λ = 1) by replacing oxygen with N_2_. A flame temperature of 2000 °C was selected to identify a high possible release (i.e., volatilization) of the ash-forming elements. It is well established in the literature that the fuel particles can experience relatively higher temperatures inside the flame compared to the furnace temperature (i.e., flue gas temperature/wall temperature).^18,24^ According to Chen et al.,^25^ the local temperature around the biomass particles inside the flame may exceed a temperature of 2000 °C. Additionally, the high temperature inside the flame facilitates the gas-phase release of fuel-inherent Si, which is particularly relevant to this study’s focus on the extraction of valuable compounds from the hot gases. Therefore, the assumption of a 2000 °C flame temperature ensures moderate to high gas-phase release of fuel inherent to Si, K, and P during the volatilization stage.

The amount of fuel-inherent elements present in the gas phase during the release calculations (A, Figure 1) at 2000 °C was taken as input for the subsequent calculations. The cooling calculations were performed using the target phase precipitation method, which focuses on the precipitation of slag/melt and the condensation of volatilized species into solid compounds at different temperatures. In this approach, a temperature range and cooling step size are specified, and phases formed at a temperature are dropped from the total mass balance.^26^ This method is particularly useful for understanding the behavior of ash-forming elements in the gas phase as they transition to condensed/precipitated phases under cooling conditions. The gas cooling was evaluated in three different atmospheres, i.e., combustion with stoichiometric amounts of air (79% N_2_, 21% O_2_; λ = 1), gasification with substoichiometric amounts of air (λ = 0.4), and pyrolysis with N_2_. The molar gas-to-fuel ash ratio during the gas cooling calculations was around 100 for rice husks and grass and around 340 for BSG. This molar ratio corresponds to the stoichiometric amount of air required for complete combustion, which was then replaced with additional N_2_ addition to achieve air gasification (λ = 0.4) and pyrolysis atmosphere to provide a similar gas-to-fuel ash ratio for each condition. The flue gas temperature of 1600 °C was selected inside the furnace. This temperature of 1600 °C was selected to model conditions representative of high-temperature industrial combustion systems while allowing for a significant margin between the expected condensation temperature of K- and P-containing gaseous species, which is around 1200 °C. This assumption can help to investigate the gas-phase behavior of these elements at higher temperatures (e.g., slagging behavior). Furthermore, the flue gas temperature of 1600 °C inside the furnace serves as a plausible gradient between the furnace and flame temperature (i.e., 2000 °C).

The transformation of volatilized species into precipitated slag/melt and condensed compounds was modeled by two different approaches. One approach focuses on modeling the condensation of the volatilized species on a deposition surface with a fixed temperature placed inside or around the flame near the burner zone (i.e., B1, Figure 1). Model approach B1 includes a gas-phase release calculation in the pyrolysis atmosphere, followed by the gas cooling calculation in combustion, gasification, and pyrolysis. This approach (B1, Figure 1) aims to identify the formed phases at different deposition surface temperatures if the gas cooling is done directly on the deposition surface. The other model approach focuses on the condensation of gaseous species in the heat exchanger zone (B2, Figure 1). During approach B2, the volatilized species from the flame react with the furnace atmosphere, and the elements retained in the gas phase during this stage were then condensed in the heat exchanger zone. Approach B2 requires one additional intermediate calculation step before the cooling step as the released gaseous species from the flame experience the reactor atmosphere. Subsequently, the available gaseous species leaving the reactor and entering the heat exchanger zone are cooled (B2, Figure 1) in combustion and gasification atmospheres. The second cooling approach aims to identify the formation of different phases in the heat exchanger zone if the gas is cooled in the heat exchanger zone, either in a combustion or gasification atmosphere.

## 3 Results and Discussion

### 3.1 Gas-Phase Release (A)

**Figure 2:**
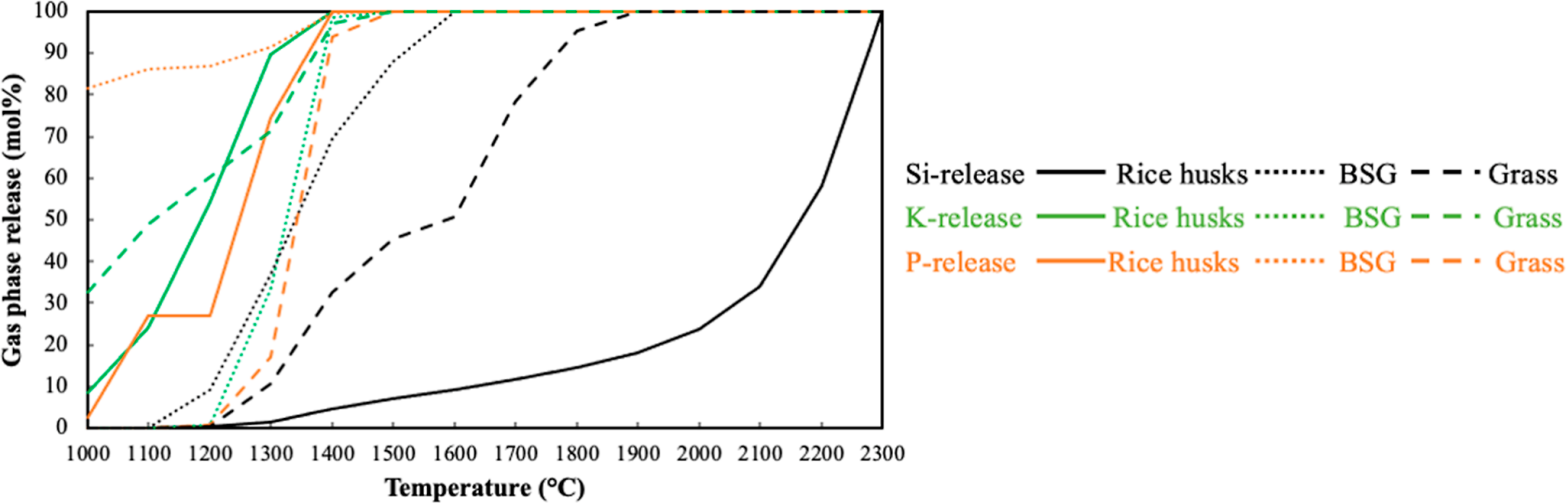
Predicted gas-phase release of ingoing Si, K, and P under a pyrolysis atmosphere between 1000 and 2300 °C.

The first step in the calculation was to determine the influence of the temperature and fuel ash composition on the gas-phase release of Si, K, and P. The pyrolysis atmosphere was selected for the gas-phase release/volatilization calculations because the fuel particles are primarily exposed to pyrolysis conditions inside the flame during entrained flow conversion. The share of the released ash-forming elements in the pyrolysis atmosphere determines the quality and yield of the subsequently condensed phases. Figure 2 shows the share of fuel-inherent Si, K, and P released to the gas phase under a pyrolysis atmosphere between 1000 and 2300 °C.

Si in all the fuels was predicted to be initially released as SiO(g) up to 2000 °C. At higher temperatures (i.e., >2000 °C), the fuel-inherent Si was released as elemental Si(g) with a small share of SiC(g). Equilibrium calculations indicated that the initial Si-release started at approximately 1100 °C for BSG and 1200 °C for rice husks and grass. Complete volatilization of Si from BSG fuel was predicted to occur around 1600 °C, at which point approximately 50% and 10% of Si had been released from grass and rice husks, respectively. This variation can be attributed to the differing Si content in each fuel and Si-containing compounds in the condensed phases. In the case of rice husks, Si was predicted to be incorporated into slag and Si-rich stable compounds such as SiO_2_ (s), Si_2_N_2_O (s), Si_3_N_4_ (s), and SiC (s). For grass and BSG, Si was predominantly retained as slag and Ca-/Mg-silicates (s). These results suggest that the formation of stable phases can also influence the Si volatilization behavior. Overall, the volatilized amount of fuel-inherent Si at a given temperature is comparable across all fuels under a pyrolysis atmosphere.

For all fuels, the K-release was predicted as KCl(g) up to 1400 °C and at higher temperatures K(g) and KCN(g) after complete devolatilization of Cl from the fuel. The predicted K-release was almost linear for rice husks and showed full devolatilization of K at 1300 °C. The initial K-release temperature for BSG was around 1200 °C. TECs indicated that the higher P content in the BSG compared to other fuels delayed K-release as most of the K was retained as K–Mg-phosphates up to 1200 °C. Full volatilization of fuel-inherent K from the grass fuel was predicted around 1350 °C. Grass fuel has the highest K and Cl content compared to other fuels. Consequently, a higher KCl(g) formation was predicted for grass. Johansen et al.^27,28^ studied the K-release behavior of corn stover by TECs under a pyrolysis atmosphere up to 1200 °C and observed the K-release as KCl(g) and K(g) up to 1200 °C. They also reported that the high-temperature release of K after complete devolatilization of Cl (i.e., via KCl(g) formation) is governed either by the thermal decomposition of carbonates or release from the silicate melt, depending on the availability of Si in the fuel. Overall, TECs predicted complete volatilization of fuel-inherent K for all of the fuels around 1350 °C.

P-release was mainly observed as P(g) and CHP(g) during the calculations. The predicted P-release for rice husks was identified as CHP(g) in the given temperature range. TECs predicted full volatilization of P present in the rice husks at around 1340 °C. For BSG, P was predicted to be released as elemental P(g) and CHP(g). With increasing temperature, the share of P(g) decreases, while the share of CHP(g) increases. The calculation results indicate that P exhibits a tendency to react with K and Mg up to 1200 °C. Meanwhile, the fuel-inherent Ca was predicted to be retained in slag and solid solution models (e.g., CAS (CaS), WOLL ((Ca,Mg)_2_Si_2_O_6_), and CLIN (Ca,Mg)SiO_3_) up to 1300 °C. The P/(K + Mg) molar ratio for rice husks, BSG, and grass is 0.31, 1.92, and 0.18, respectively. It is more likely that the relatively higher share of P was released at lower temperatures from the BSG fuel due to a surplus of P compared to other fuels, where P-release was predicted to be around 80% at 1000 °C and 100% at around 1300 °C. For grass, the initial P-release was predicted to be around 1200 °C. The high share of K in grass facilitates the formation of KMgPO_4_ (s) up to 1200 °C. Overall, the P-release of each fuel was predicted to be 100% around 1500 °C.

### 3.2 Cooling on a Deposition Surface inside or around the Flame near the Burner Zone (B1)

#### 3.2.1 Rice Husks

**Figure 3:**
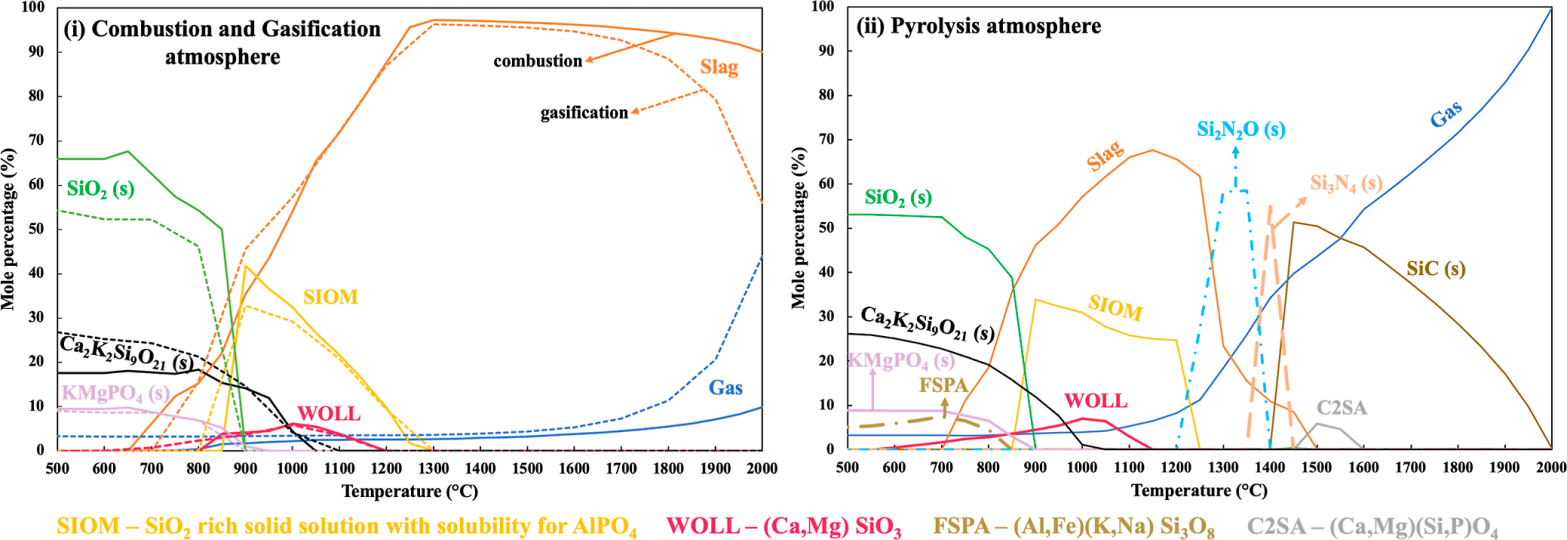
Predicted distribution of ash-forming elements in gas and condensed phases between 500 and 2000 °C under different atmospheres for rice husks—gas cooling on a deposition surface inside (i.e., ii, pyrolysis) or around (i.e., i, combustion/gasification) the flame near the burner zone. The calculated results are obtained by target phase precipitation calculations at different fixed temperatures.

Figure 3 represents the gas cooling results obtained from target phase precipitation calculations for rice husks. The graphs illustrate the predicted distribution of gas and condensed compounds between 500 and 2000 °C. Based on the release calculation (i.e., Section 3.1), the ash-forming elements present in the predicted gaseous species at 2000 °C primarily consisted of Si (≈67 mol %), with minor amounts of Ca, K, Mg, P, S, and Cl. The molar ratio of Si to other ash-forming elements in the released gas stream at 2000 °C was around 2.7.

The calculation results obtained during combustion and gasification atmosphere indicate that the ash-forming elements were primarily incorporated in slag containing a Si-rich oxide melt (slag) at cooling/surface temperatures from 2000 to 1300 °C, with minor quantities remaining in the gas phase. The predicted slag during combustion and gasification atmosphere was rich in Si with minor amounts of K, Ca, Mg, and P. A decrease in the cooling/surface temperature showed a reduction in the slag formation with an increase in the formation of solid-phase compounds and solid-solution models. The predicted solid compounds and solution models were identified as SIOM (SiO_2_-rich solid solution with solubility for AlPO_4_) around 1300 °C, Ca_2_K_2_Si_9_O_21_ (s) around 1000 °C, and KMgPO_4_ (s) and SiO_2_ (s) around 900 °C with small amounts of WOLL (i.e., (Ca,Mg)SiO_3_).

During the pyrolysis cooling conditions, a relatively higher share of ash-forming elements was retained in the gas phase at high cooling/surface temperatures (i.e., >1500 °C) compared to the combustion and gasification cooling conditions. Additionally, TECs predicted slag formation between 1500 and 700 °C, where the slag contained a Si-rich oxide melt with minor amounts of K, Ca, Mg, and P. The predicted solid compounds and solution models during pyrolysis cooling were dominated by Si-containing species throughout the entire temperature range. At high cooling/surface temperatures, TECs predicted the formation of SiC(s) between 2000 and 1500 °C when a significant amount of Si in the gas phase was predicted to be deposited as SiC(s). Additionally, a small share of C2SA ((Ca,Mg)(Si,P)O_4_) was also predicted to be deposited around 1500 °C. The formation of Si–N-containing compounds (i.e., Si_3_N_4_ and Si_2_N_2_O) was predicted between 1450 and 1200 °C. In previous experiments, the formation of SiC (s) and Si_3_N_2_O (s) was reported during the pyrolysis of rice husks between 1200 and 1500 °C in a lab-scale fixed-bed setup.^29,30^ A decrease in the cooling/surface temperatures resulted in the formation of SIOM around 1250 °C, Ca_2_K_2_Si_9_O_21_ (s) around 1100 °C, and KMgPO_4_ (s) and SiO_2_ (s) around 900 °C with small amounts of WOLL (i.e., (Ca,Mg)SiO_3_) and FSPA ((Al,Fe)(K,Na)Si_3_O_8_).

#### 3.2.2 Brewer’s Spent Grains

**Figure 4:**
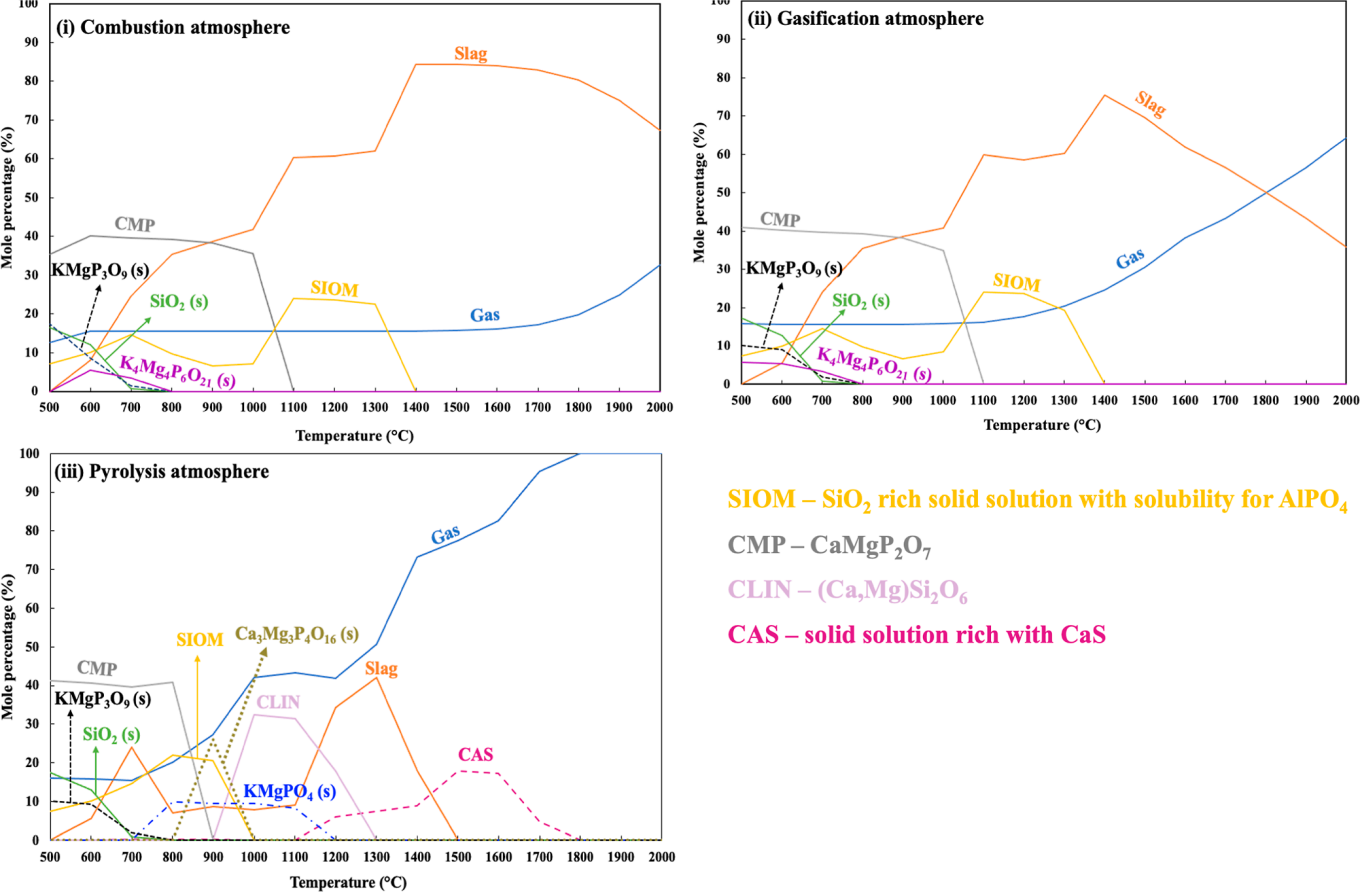
Predicted distribution of ash-forming elements in gas and condensed phases between 500 and 2000 °C under different atmospheres for BSG—gas cooling on a deposition surface inside (i.e., iii, pyrolysis) or around (i.e., i and ii, combustion/gasification) the flame near the burner zone. The calculated results are obtained by target phase precipitation calculations at different fixed temperatures.

Figure 4 shows the results obtained by model approach B1 for BSG fuel. The ash-forming elements present in the released gaseous species at 2000 °C (i.e., from Section 3.1) were dominated by Si + P (≈55 mol %) with moderate to minor amounts of Ca, Mg, S, K, and Cl. In the combustion and gasification cooling atmosphere, most of the ash-forming elements formed slag between 2000 and 500 °C, and only a moderate amount of ash-forming elements (i.e., primarily S, Cl, and some P) were predicted to be retained in the gas phase. The gasification atmosphere displayed a relatively higher share of P retained in the gas phase above 1500 °C compared with the combustion atmosphere. The predicted slag above 1400 °C and below 1000 °C mainly contained a Ca–Mg-rich phosphosilicate melt with a minor amount of K. TECs predicted the formation of a SiO_2_-rich solid solution with solubility for AlPO_4_ (SIOM) around 1400 °C. Consequently, the predicted slag was composed of a Ca–Mg-rich phosphate melt with a minor amount of K between 1000 and 1400 °C. The rest of the solid compounds and solid solution model were predicted as CMP (CaMgP_2_O_7_) around 1100 °C and K_4_Mg_4_P_6_O_11_ (s), KMgP_3_O_9_ (s), and SiO_2_ (s) around 800 °C.

In the pyrolysis cooling atmosphere, most of the released ash-forming elements were retained in the gas phase between 2000 and 1500 °C. TECs also predicted slag formation between 1500 and 500 °C, where a moderate amount of the ash-forming elements was incorporated into the slag. The predicted slag was identified as a Ca–Mg-rich phosphosilicate melt with a minor share of K. At high cooling/surface temperatures, i.e., around 1800 °C, TECs predicted the formation of a solid solution rich in CaS (CAS). P-containing solid compounds and solution models were predicted to be condensed as KMgPO_4_(s) around 1200 °C, Ca_3_Mg_3_P_4_O_16_(s) around 1000 °C, CMP (CaMgP_2_O_7_) around 900 °C, and KMgP_3_O_9_(s) around 800 °C. In addition to the P-containing compounds, Si-containing solid compounds and solid solution models were predicted to be formed as CLIN ((Ca,Mg)Si_2_O_6_) around 1300 °C, SIOM around 1000 °C, and SiO_2_(s) around 800 °C. The most likely reason for the predicted formation of CLIN around 1300 °C could be lower slag formation compared to the other two conditions, which means increased availability of Ca and Mg in the gas phase that can subsequently react with the Si to form solid compounds.

#### 3.2.3 Grass

**Figure 5:**
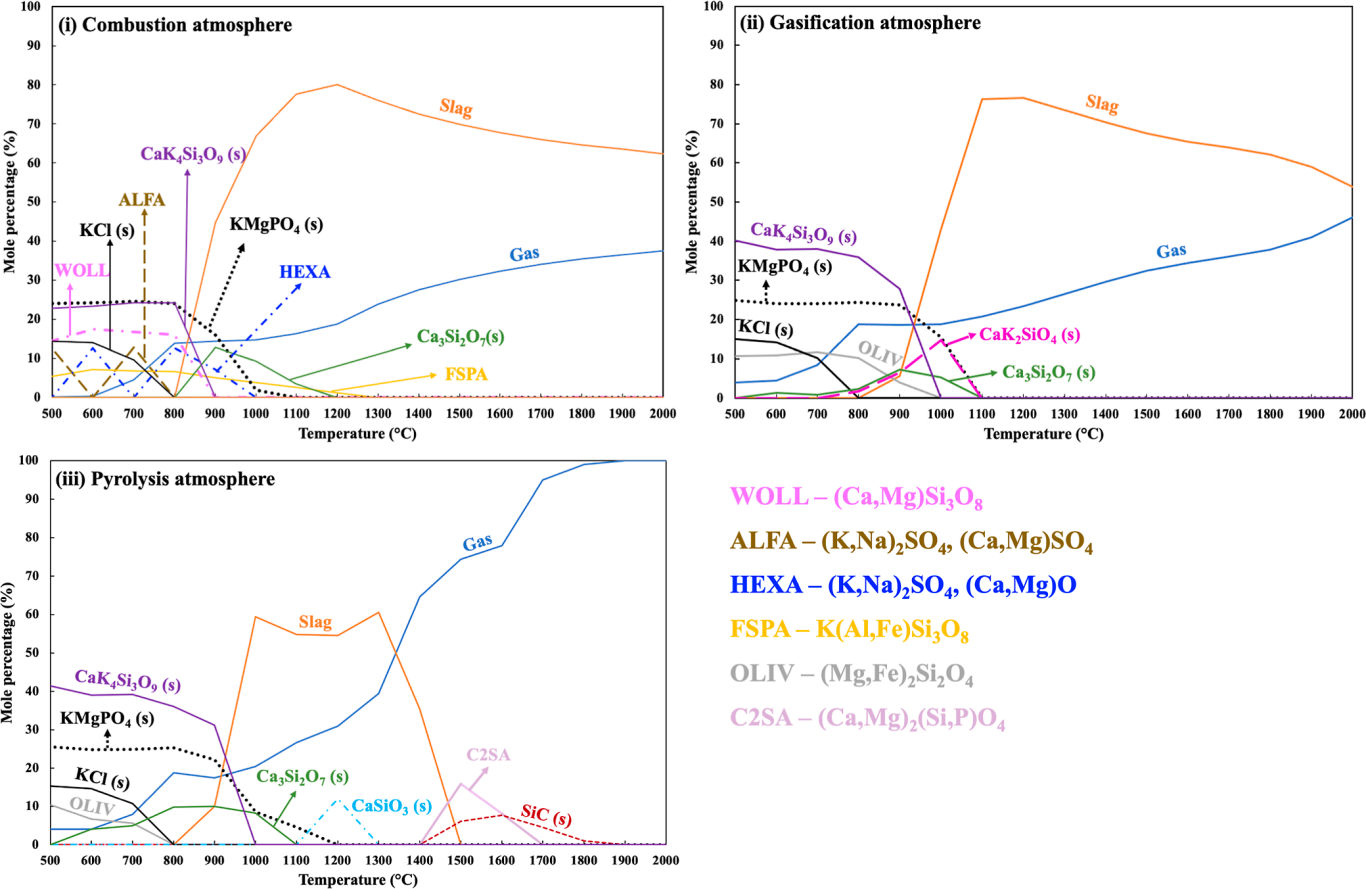
Predicted distribution of ash-forming elements in gas and condensed phases between 500 and 2000 °C under different atmospheres for grass—gas cooling on a deposition surface inside (i.e., iii, pyrolysis) or around (i and ii, combustion/gasification) the flame near the burner zone. The calculated results are obtained by target phase precipitation calculations at different fixed temperatures.

The predicted distribution of gas and condensed phases at different cooling/surface temperatures obtained by model approach B1 is presented in Figure 5. For the grass fuel, the ash-forming elements present in the released gaseous stream at 2000 °C from the previous calculation (i.e., Section 3.1) were dominated by K + Si (≈56 mol %) with moderate to minor amounts of Ca, Mg, P, S, and Cl.

The combustion and gasification cooling profiles indicate that most of the ash-forming elements in the gaseous stream formed slag from 2000 to 1200 °C. The predicted slag contained a K-, Ca-, and Mg-rich phosphosilicate melt, where all the released Ca, Mg, P, and Si and a moderate amount of K from the gaseous stream were incorporated into the slag. The share of Si in the slag decreases with a decreasing cooling/surface temperature. The remaining K and all of the S and Cl were retained in the gas phase. During the combustion atmosphere, solid compounds and solution models were predicted to form below 1300 °C. The predicted solid compounds and solution models during the combustion atmosphere were FSPA (feldspar, K(Al, Fe)Si_3_O_8_), Ca-silicates (s), WOLL ((Ca, Mg)SiO_3_), Ca–K-silicates (s), K–Mg-phosphate (s), HEXA ((K, Na)_2_SO_4_, (Ca, Mg)O), ALFA ((K, Na)_2_SO_4_, (Ca, Mg)SO_4_), and KCl (s). The predicted share of K–Ca-silicates (s) was increased in the gasification atmosphere compared to the combustion atmosphere. Additionally, the gasification atmosphere indicated a higher retention of S below 1000 °C. Consequently, the solid solution models HEXA and ALFA did not form under a gasification atmosphere. An additional formation of OLIV (olivine, (Mg, Fe)_2_SiO_4_) was predicted under a gasification atmosphere.

The calculations performed under a pyrolysis cooling atmosphere predicted a high retention of ash-forming elements from 2000 to 1500 °C. TECs predicted slag formation between 1500 and 800 °C, where the slag was composed of a K-, Ca-, and Mg-rich phosphosilicate melt. At high cooling/surface temperatures (i.e., >1500 °C), TECs indicated the formation of SiC (s) around 1900 °C and C2SA ((Ca, Mg)_2_(Si, P)O_4_) around 1700 °C. The rest of the solid compounds were predicted to be formed as Ca-silicates (s), Ca–K-silicate (s), OLIV, K–Mg-phosphate (s), and KCl (s) below 1300 °C.

### 3.3 Cooling in the Heat Exchanger Zone (B2)

#### 3.3.1 Rice Husks

The intermediate calculation performed under combustion and gasification atmospheres indicated that all of the ash-forming elements except S and Cl were incorporated into a slag at 1600 °C. The predicted slag was enriched in an oxide melt (i.e., Si_2_O_4_) with minor amounts of K, Ca, Mg, and P. During the release calculation (i.e., Section 3.1), TECs predicted the fuel-inherent Si-release as SiO(g). According to the intermediate calculation, the equilibrium conditions do not favor the stabilization of the SiO(g) in the reactor atmosphere (i.e., combustion and gasification). However, nonkinetic parameters like short residence time could affect the outcome in real operational conditions. In general, TECs did not predict the retention of Si, K, and P in the gas stream entering the heat exchanger zone. Consequently, the formation of valuable Si- and K–P-containing compounds in the heat exchanger zone after being exposed to a combustion and gasification reactor atmosphere was not possible in the case of rice husks.

#### 3.3.2 Brewer’s Spent Grains

The intermediate calculation suggests that all of the gaseous K, Si, and P generated during the release calculation can form slag in the reactor during the combustion atmosphere. However, the calculation performed under a gasification atmosphere indicates that 24 mol % of the total P released to the gas phase during the release calculation (i.e., Section 3.1), along with Na, Zn, S, and Cl, was retained in the gas phase following exposure to the combustion and gasification reactor atmosphere. During the gasification atmosphere, the ash-forming elements present in the gaseous stream entering the heat exchanger zone mainly contained S + P (≈91 mol %) with minor amounts of Zn and Cl.

**Figure 6:**
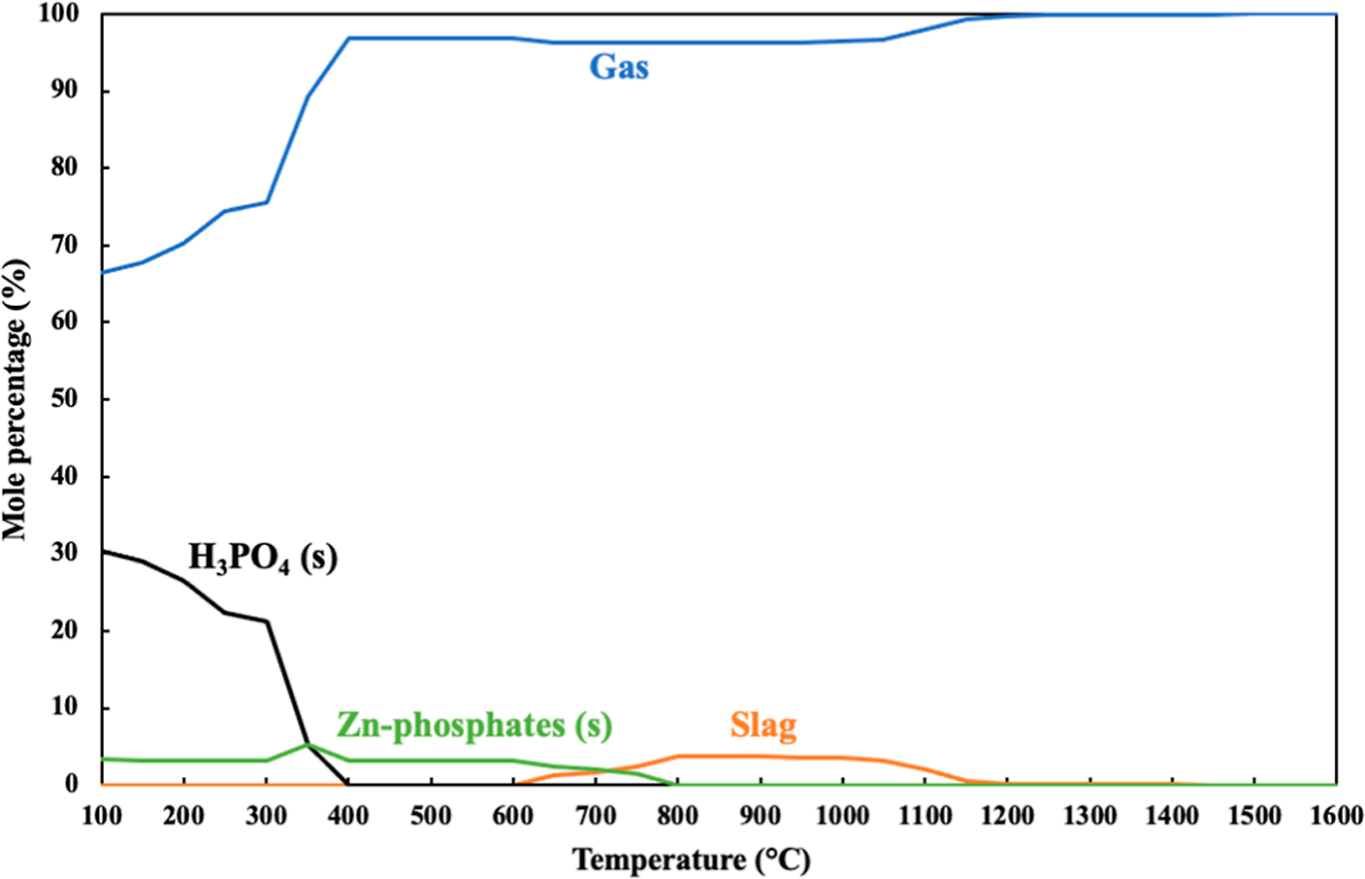
Predicted distribution of ash-forming elements in gas and condensed phases between 100 and 1600 °C under gasification atmospheres for BSG—gas cooling results obtained by target phase precipitation calculations in the heat exchanger zone.

These gaseous species were then cooled in the gasification atmosphere by approach B2, and the distribution of predicted gas and condensed compounds in the heat exchanger zone can be seen in Figure 6. A major share of ash-forming elements in the heat exchanger zone were retained in the gas phase between 1600 and 400 °C. A minor amount of slag containing a Zn-phosphate melt was predicted to form between 1200 and 600 °C. The predicted solid compounds were identified as Zn-phosphates around 800 °C and H_3_PO_4_ (s) around 400 °C. Almost 90% of P entering the heat exchanger zone was predicted to be condensed as H_3_PO_4_ (s) starting at 400 °C. In contrast, all of the S and Cl were retained in the gas phase.

#### 3.3.3 Grass

The results obtained from the intermediate calculations suggest that most of the ash-forming elements under equilibrium conditions were associated with the slag at 1600 °C, except for parts of K, Na, S, and Cl. The only difference between combustion and gasification conditions was the share of K in the gas stream at 1600 °C. The predicted share of K within the ash-forming elements present in the gas stream during combustion and gasification conditions at 1600 °C is 57 and 62 mol % of the total K released during the previous calculation (i.e., Section 3.1), respectively. During both combustion and gasification in the reactor atmosphere at 1600 °C, the intermediate calculation indicated that the gas stream entering the heat exchanger zone mainly contained K and Cl with minor amounts of Na and S.

The gas cooling calculation in the heat exchanger zone mainly predicted the formation of K, sulfate (s), K_2_CO_3_ (s), and KCl (s) below 1000 °C under combustion and gasification atmospheres. Therefore, TECs did not indicate the possibility of forming valuable Si- and K–P-containing compounds in the heat exchange zone.

### 3.4 General Discussion and Practical Implications

The primary goal for obtaining valuable Si- and K–P-containing compounds from the gas phase is to achieve high gas-phase release of the targeted elements in the flame during the volatilization stage. The fuel particle size and burner configuration are critical factors influencing the gas-phase release of ash-forming elements in the flame. For instance, Wang et al.^31^ reported a relatively high share of Si in the PM1 fraction with smaller fuel particles (*d*_50_: ≈20 μm) compared to the larger fuel particles (*d*_50_: ≈140 μm) during the entrained combustion of rice husks around 1200 °C, likely due to increased surface area and higher temperatures leading to faster volatilization rates inside the flame. Additionally, small fuel particles can also provide high flame stability and fewer fluctuations,^32^ which can help to achieve a uniform temperature profile and longer residence time for fuel particles in the flame. These results indicate that a high volatilization of ash-forming elements can be achieved by employing small fuel particle sizes. Göktepe et al.^33^ demonstrated that acoustic forcing in a 150 kW swirl biomass powder burner enhanced the heat release rate at low-frequency excitation, improving particle dispersion inside the flame. This improved dispersion can reduce the mutual interaction between ash-forming elements inside the flame and increase the degree of volatilization. As a result, this enhances the potential for the formation of the targeted compounds.

Two different gas cooling approaches were studied in this work. In practice, the gas cooling on a deposition surface with a certain temperature near the burner zone (i.e., approach B1, Section 3.2) could be done by placing a fixed-temperature deposition surface inside or around the flame near the burner zone. Placing a deposition surface inside the flame would enable condensation at a target temperature in a pyrolysis environment. Cooling in the combustion or gasification atmosphere near the burner zone (i.e., around the flame) could be achieved by placing the deposition surface around the flame. The local environment around the flame is highly dependent on the flame’s size and shape. Therefore, this approach would require an additional understanding of the flame pattern. The gas cooling can also be in the heat exchanger zone (i.e., approach B2, Section 3.3) after the gaseous stream reacts with the combustion/gasification atmosphere ahead of the flame zone. A part of the released gaseous species in this approach could either be deposited on the char particles or form slag, depending on the fuel ash composition. Moreover, the gaseous species reaching the heat exchanger zone may change the formation pathways of condensed compounds as the temperature of the heat exchanger surface will be changed once a layer of solid particles is formed.

Rice husks containing a significant share of Si in the fuel could be one of the fuels to be employed in entrained flow systems regarding Si extraction as they possess a very low share of other ash-forming elements. During the pyrolysis/volatilization conditions, the initial Si-release was predicted to be around 1200 °C (see Figure 2), where the fuel-inherent Si was primarily released as SiO (g). A previous study conducted on pyrolysis of rice husks in a lab-scale drop tube furnace showed a minor volatilization of Si (<1%) at 1200 and 1450 °C.^21^ Moreover, TECs showed that only a moderate amount of Si (i.e., 25%) is volatilized to the gas phase during pyrolysis/volatilization conditions, even at a temperature as high as 2000 °C (see Figure 2). The combined results from this study and previous experimental investigations in the drop tube furnace^21^ suggest that very high temperatures during the volatilization stage are necessary to get significant volatilization of Si from the fuel. The gas cooling calculations performed on rice husks only predicted the formation of valuable and relatively pure Si-containing compounds as SiC(s) and Si–N compounds (s) (ii, Figure 3) around 1500 °C under a pyrolysis cooling atmosphere when the gas cooling was modeled in the burner/flame zone (i.e., approach B1, Section 3.2). One potential approach to achieving temperatures around 1500 °C is preheating the air or inlet gas stream. Overall, attaining these cooling conditions in practical settings poses challenges.

BSG containing a high share of P and Si with moderate to minor amounts of Ca, Mg, and K showed complete volatilization of all of the ash-forming elements at 2000 °C under pyrolysis/volatilization conditions (see Figure 2). During the gas cooling calculations, TECs did not predict the formation of pure Si- and K–P-containing compounds inside or around the flame near the burner zone (i.e., approach B1, Figure 4). TECs predicted that the majority of P was retained in the slag (i.e., Ca–Mg-rich phosphosilicate melt) between 1000 and 1600 °C inside or around the flame, near the burner zone and in the furnace zone. This behavior was confirmed in experimental work by Pachchigar et al.,^22^ where a significant fraction of P was retained in the Ca–Mg-rich phosphosilicate melt during entrained flow combustion of BSG at 1200 and 1450 °C. This comparison demonstrates the consistency between TECs’ predictions and experimental observations. In general, the gas cooling performed near the burner zone indicates a higher possibility of forming Ca-bearing phosphates than that of K-bearing phosphates (Figure 4). The lower gas-phase release of Ca and Mg compared to other ash-forming elements could increase the share of K-bearing phosphates. This can be achieved by lowering flame temperatures, which may facilitate the retention of Ca and Mg in the char, thereby increasing the probability of obtaining pure K-bearing phosphates near the burner zone. In contrast, the formation of relatively pure H_3_PO_4_ (s) in the heat-exchanger zone was predicted under gasification reactor conditions at temperatures lower than 400 °C (i.e., approach B2, Figure 6). The entrained flow combustion experiments with BSG at 1200 and 1450 °C reported that the PM1 fraction was dominated by P with moderate to minor amounts of K, S, and Cl.^22^ Consequently, the surplus of P compared to Si and cations in the BSG fuel may facilitate the formation of pure P-containing compounds in the heat-exchanger zone at lower surface temperatures (i.e., <400 °C). The formation of P_*x*_O_*y*_(g) from the furnace under gasification conditions with enough partial steam pressure may enhance the possibility of forming H_3_PO_4_(s) in the heat exchanger zone. This suggests that BSG fuel could be beneficial for targeted P-recovery, particularly in downstream cooling zones.

For grass fuel (i.e., K–Si-rich with moderate amounts of Ca, Mg, and P), TECs did not indicate the probability of extracting pure Si- and K–P-containing compounds. According to the TECs, a high share of K and Si in the released gaseous species led to the formation of a K–Ca–Mg-rich phosphosilicate melt when reacted under combustion and gasification atmospheres. This result agrees with a previous study conducted on the entrained flow combustion of grass at 1200 and 1450 °C,^22^ where the majority of retained K, Ca, and Si within the resulting coarse ashes were found as a similar K–Ca–Mg-rich phosphosilicate melt. The formation of these melts poses significant challenges for extracting valuable Si- and K–P-containing compounds as the retention of these elements into the melt reduces the volatile share and their subsequent potential for condensation.

In general, TECs are based on several assumptions such as complete mixing of reactants and no limitations to the reaction time. These assumptions do not fully capture the nonequilibrium conditions typically encountered in practical entrained flow conversion systems. In practice, several nonequilibrium parameters, such as the local atmosphere inside the flame, particle dispersion, reaction rate, and deposition surface conditions, could influence the possibilities of forming the targeted compounds. Despite these restrictions, TECs provide valuable insights into the thermodynamic driving force governing the volatilization of ash-forming elements and the potential formation of valuable Si- and K–P-containing compounds during the thermal conversion of agricultural biomass in entrained flow conditions. This information is crucial for identifying optimal conditions that enhance the extraction of valuable targeted compounds from the gases generated during the process via subsequent condensation, providing a guiding tool for both experimental design and industrial application.

## 4 Conclusion

The potential of extracting valuable Si and K–P compounds via subsequent condensation from the gases in entrained flow conditions was analyzed by performing thermodynamic equilibrium calculations on three types of agricultural biomass with different ash compositions, i.e., rice husks (Si-rich), BSGs (P–Si-rich with moderate to minor amounts of Ca, Mg, and K), and grass (K–Si-rich with moderate Ca, Mg, and P).

The initial gas-phase Si-release of each fuel was identified at around 1200 °C. The gas-phase release of Si was mainly influenced by the retention of fuel-inherent Si in stable phases, such as Si-rich compounds (i.e., SiO_2_ (s), Si_2_N_2_O (s), Si_3_N_4_ (s), and SiC (s)) for rice husks and Ca-/Mg-silicates (s) for grass and BSG. The initial temperature for the gas-phase release of P and K was dependent on the fuel ash composition, and an excess amount of either P or K in the fuel ash could delay the gas-phase release of one another.

The predicted pure solid compounds formed on the deposition surface inside the flame indicate the potential separation of SiC (s) from the K–P-containing gases at high surface temperatures (i.e., >1500 °C) for rice husks and grass fuel. A high molar ratio of Si/P and pyrolysis cooling conditions facilitate separation during the condensation of solid Si compounds and K–P compounds. The condensed K-bearing phosphates inside and around the flame near the burner zone were identified as KPMgO_4_ (s) (for all the fuels), KMgP_3_O_9_ (s), and K_4_M_g_P_6_O_21_ (s) (for BSG). For BSG, the formation of K–Mg-phosphates inside and around the flame near the burner zone was predicted along with Ca–Mg-phosphates and SiO_2_ (s) from temperatures below 1200 °C. In the case of grass, the formation of K–Mg-phosphates was predicted with Ca–K-silicates (s), Ca-silicates (s), and KCl (s) inside and around the flame near the burner zone.

The gas cooling approach in the heat exchanger zone predicted most of the gas-phase Si-, K-, and P-containing compounds precipitated as slag after interacting with the furnace atmosphere. Therefore, not enough gaseous species are available in the heat exchanger zone to form valuable Si and K–P compounds. However, this approach indicates the potential for extracting relatively pure H_3_PO_4_ (s) for BSG in the heat exchanger zone.

The results from the thermodynamic equilibrium calculations suggest that the fuel composition, cooling atmosphere, and positioning of the collection unit/probe highly influence the deposition of Si and K–P compounds on the cooled surfaces. The qualitative results obtained by this study shall be taken into practice to identify the potential extraction of relatively pure and valuable Si- and K–P-containing compounds.

## References

[ref1] Zevenhoven M.; Yrjas P.; Skrifvars B. J.; Hupa M. Characterization of Ash-Forming Matter in Various Solid Fuels by Selective Leaching and Its Implications for Fluidized-Bed Combustion. Energy Fuels 2012, 26 (10), 6366–6386. 10.1021/ef300621j.

[ref2] Hedayati A.; Lindgren R.; Skoglund N.; Boman C.; Kienzl N.; Öhman M. Ash Transformation during Single-Pellet Combustion of Agricultural Biomass with a Focus on Potassium and Phosphorus. Energy Fuels 2021, 35 (2), 1449–1464. 10.1021/acs.energyfuels.0c03324.

[ref3] Boström D.; Skoglund N.; Grimm A.; Boman C.; Öhman M.; Broström M.; Backman R. Ash Transformation Chemistry during Combustion of Biomass. Energy Fuels 2012, 26 (1), 85–93. 10.1021/ef201205b.

[ref4] Fürsatz K.; Fuchs J.; Benedikt F.; Kuba M.; Hofbauer H. Effect of Biomass Fuel Ash and Bed Material on the Product Gas Composition in DFB Steam Gasification. Energy 2021, 219, 119650 10.1016/j.energy.2020.119650.

[ref5] Kleinhans U.; Wieland C.; Frandsen F. J.; Spliethoff H. Ash Formation and Deposition in Coal and Biomass Fired Combustion Systems: Progress and Challenges in the Field of Ash Particle Sticking and Rebound Behavior. Prog. Energy Combust. Sci. 2018, 68, 65–168. 10.1016/j.pecs.2018.02.001.

[ref6] Tissari J.; Sippula O.; Kouki J.; Vuorio K.; Jokiniemi J. Fine Particle and Gas Emissions from the Combustion of Agricultural Fuels Fired in a 20 KW Burner. Energy Fuels 2008, 22 (3), 2033–2042. 10.1021/ef700766y.

[ref7] Lindström E.; Sandström M.; Boström D.; Öhman M. Slagging Characteristics during Combustion of Cereal Grains Rich in Phosphorus. Energy Fuels 2007, 21 (2), 710–717. 10.1021/ef060429x.

[ref8] Morris J. D.; Daood S. S.; Nimmo W. Agglomeration and the Effect of Process Conditions on Fluidized Bed Combustion of Biomasses with Olivine and Silica Sand as Bed Materials: Pilot-Scale Investigation. Biomass Bioenergy 2020, 142, 105806 10.1016/j.biombioe.2020.105806.

[ref9] Yuan M.; Guo X.; Liu Y.; Pang H. Si-Based Materials Derived from Biomass: Synthesis and Applications in Electrochemical Energy Storage. J. Mater. Chem. A 2019, 7 (39), 22123–22147. 10.1039/C9TA06934H.

[ref10] Zawrah M. F.; Zayed M. A.; Ali M. R. K. Synthesis and Characterization of SiC and SiC/Si 3N 4 Composite Nano Powders from Waste Material. J. Hazard. Mater. 2012, 227–228, 250–256. 10.1016/j.jhazmat.2012.05.048. 22673059

[ref11] Rajasekar M.; Associate R.; Nandhini U.; Balakrishnan K. A Review on Role of Macro Nutrients on Production and Quality of Vegetables. Int. J. Chem. Stud. 2017, 5 (3), 304–309.

[ref12] Belov S. V.; Danyleiko Y. K.; Glinushkin A. P.; Kalinitchenko V. P.; Egorov A. V.; Sidorov V. A.; Konchekov E. M.; Gudkov S. V.; Dorokhov A. S.; Lobachevsky Y. P.; Izmailov A. Y. An Activated Potassium Phosphate Fertilizer Solution for Stimulating the Growth of Agricultural Plants. Front. Phys. 2021, 8, 618320 10.3389/fphy.2020.618320.

[ref13] Falk J. The fate and ash transformations of phosphorus in combustion of biomass and sewage sludge, Doctoral Thesis, ISBN: 978-91-8048-169-4, Luleå University of Technology, 2022.

[ref14] Liu Y.; Guo Y.; Zhu Y.; An D.; Gao W.; Wang Z.; Ma Y.; Wang Z. A Sustainable Route for the Preparation of Activated Carbon and Silica from Rice Husk Ash. J. Hazard. Mater. 2011, 186 (2–3), 1314–1319. 10.1016/j.jhazmat.2010.12.007. 21194835

[ref15] Mananda A. B.; Harada H.; Halem H. I. A.; Mitoma Y.; Keiko F. Study of Potassium Recovery from Biomass Ash Using Tartaric Acid and Syngenite Method. J. Mater. Sci. Chem. Eng. 2021, 09 (05), 39–52. 10.4236/msce.2021.95004.

[ref16] Tan Z.; Lagerkvist A. Phosphorus Recovery from the Biomass Ash: A Review. Renew. Sustain. Energy Rev. 2011, 15 (8), 3588–3602. 10.1016/j.rser.2011.05.016.

[ref17] Eriksson G.; Grimm A.; Skoglund N.; Boström D.; Öhman M. Combustion and Fuel Characterisation of Wheat Distillers Dried Grain with Solubles (DDGS) and Possible Combustion Applications. Fuel 2012, 102, 208–220. 10.1016/j.fuel.2012.05.019.

[ref18] Bejarano P. A.; Levendis Y. A. Single-Coal-Particle Combustion in O2/N2 and O2/CO2 Environments. Combust. Flame 2008, 153 (1–2), 270–287. 10.1016/j.combustflame.2007.10.022.

[ref19] Qi S.; Wang Z.; Costa M.; He Y.; Cen K. Ignition and Combustion of Single Pulverized Biomass and Coal Particles in N2/O2 and CO2/O2 Environments. Fuel 2021, 283, 118956 10.1016/j.fuel.2020.118956.

[ref20] Liaw S. B.; Wu H. Importance of flue gas cooling conditions in particulate matter formation during biomass combustion under conditions pertinent to pulverized fuel applications. Proc. Combust. Inst. 2021, 38, 5201–5208. 10.1016/j.proci.2020.06.152.

[ref21] Pachchigar S.; Hannl T. K.; Öhman M. Ash Formation during Combustion of Rice Husks in Entrained Flow Conversion Conditions. Energy Fuels 2024, 38 (14), 13278–13294. 10.1021/acs.energyfuels.4c01413.

[ref22] Pachchigar S.; Hannl T. K.; Skoglund N.; Öhman M. Ash Transformation during Combustion of Agricultural Biomass in Entrained Flow Conditions with a Focus on Phosphorus. Energy Fuels 2025, 39, 1384–1400. 10.1021/acs.energyfuels.4c05064.

[ref23] Hannl T. K.; Sefidari H.; Kuba M.; Skoglund N.; Öhman M. Thermochemical Equilibrium Study of Ash Transformation during Combustion and Gasification of Sewage Sludge Mixtures with Agricultural Residues with Focus on the Phosphorus Speciation. Biomass Convers. Biorefin. 2021, 11, 57–68. 10.1007/s13399-020-00772-4.

[ref24] Jia Y.; Li Z.; Wang Y.; Wang X.; Lou C.; Xiao B.; Lim M. Visualization of Combustion Phases of Biomass Particles: Effects of Fuel Properties. ACS Omega 2021, 6 (42), 27702–27710. 10.1021/acsomega.1c02783. 34722970 PMC8552231

[ref25] Chen T.; Ku X.; Lin J.; Jin H. Modelling the Combustion of Thermally Thick Biomass Particles. Powder Technol. 2019, 353, 110–124. 10.1016/j.powtec.2019.05.011.

[ref26] Bale C. W.; Bélisle E.; Chartrand P.; Decterov S. A.; Eriksson G.; Gheribi A. E.; Hack K.; Jung I. H.; Kang Y. B.; Melançon J.; Pelton A. D.; Petersen S.; Robelin C.; Sangster J.; Spencer P.; Van Ende M. A. FactSage Thermochemical Software and Databases, 2010–2016. CALPHAD 2016, 54, 35–53. 10.1016/j.calphad.2016.05.002.

[ref27] Anca-Couce A.; Sommersacher P.; Hochenauer C.; Scharler R. Multi-Stage Model for the Release of Potassium in Single Particle Biomass Combustion. Fuel 2020, 280, 118569 10.1016/j.fuel.2020.118569.

[ref28] Johansen J. M.; Jakobsen J. G.; Frandsen F.; Glarborg P. Release of K, Cl, and S during Pyrolysis and Combustion of High-Chlorine Biomass. Energy Fuels 2011, 25 (11), 4961–4971. 10.1021/ef201098n.

[ref29] Krishnarao R. V.; Mahajan Y. R.; Kumar T. J. Conversion of Raw Rice Husks to SiC by Pyrolysis in Nitrogen Atmosphere. J. Eur. Ceram. Soc. 1998, 18 (2), 147–152. 10.1016/S0955-2219(97)00093-9.

[ref30] Gorthy P.; Mukunda Pudukottah G. Production of Silicon Carbide from Rice Husks. J. Am. Ceram. Soc. 1999, 82 (6), 1393–1400. 10.1111/J.1151-2916.1999.TB01929.X.

[ref31] Wang Y.; Wu J.; Li X.; Han J.; Yu D.; Xu M.; Wendt J. O. L. Comparison Study of Ash Partitioning and Deposition Behavior between Two Rice Husk Fuels under a 100 KW Combustor. Energy Fuels 2019, 33 (11), 11968–11975. 10.1021/acs.energyfuels.9b02914.

[ref32] Dal Belo Takehara M.; Chishty M. A.; Umeki K.; Gebart R. Pulverized Biomass Flame under Imposed Acoustic Oscillations: Flame Morphology and Emission Characteristics. Fuel Process. Technol. 2022, 238, 107484 10.1016/j.fuproc.2022.107484.

[ref33] Göktepe B.; Gebart R.; Edgar F.; Leitao N. Simultaneous Pressure and Heat Release Measurements in a 150 KW Wood Powder Burner. In SPEIC 10-Towards Sustainable Combustion, Tenerife, Spain, 2010.

